# Genome-Wide Identification of Luffa Sucrose Synthase Genes Reveals *LaSUS1*-Mediated Sugar Metabolism Boosting Drought Tolerance

**DOI:** 10.3390/ijms26125675

**Published:** 2025-06-13

**Authors:** Xiaocheng Tian, Hanyi Wang, Jianting Liu, Liujing Huang, Xiaohui Zheng, Yufeng Li, Shaolong Sun, Chongjian Ma, Hongbo Zhao, Puyan Zhao

**Affiliations:** 1 College of Horticulture, South China Agricultural University, Guangzhou 510642, China; tianxiaocheng@nwsuaf.edu.cn (X.T.); wanghanyireal@163.com (H.W.); hlj2024@stu.scau.edu.cn (L.H.); 15521022481@163.com (X.Z.); 15800014767@163.com (Y.L.); 2 Guangdong Provincial Key Laboratory of Utilization and Conservation of Food and Medicinal Resources in Northern Region, School of Biology and Agriculture, Shaoguan University, Shaoguan 512005, China; chjma@sgu.edu.cn; 3 College of Natural Resources and Environment, South China Agricultural University, Guangzhou 510642, China; sunshaolong328@scau.edu.cn; 4 Fujian Key Laboratory of Vegetable Genetics and Breeding, Crops Research Institute, Fujian Academy of Agricultural Sciences, Fuzhou 350003, China; ljt338625@163.com

**Keywords:** *Luffa acutangula*, *SUS*, drought, expression profile

## Abstract

Luffa (*Luffa acutangula*) serves as an important edible, medicinal, and industrial crop. Sucrose synthase (SUS, EC 2.4.1.13) catalyzes sucrose metabolism and facilitates the entry of photosynthetically derived sucrose into metabolic pathways, playing crucial roles in plant growth, development, and stress responses. However, systematic investigations on the *SUS* gene family in luffa remain relatively scarce. In this study, we identified nine *LaSUS* family members distributed unevenly across six chromosomes. Their physicochemical properties and evolutionary relationships were systematically elucidated using bioinformatics tools. RNA-seq analysis revealed distinct expression patterns of *LaSUS* genes during luffa fruit aging, with most genes showing significant down-regulation during this process. Notably, several genes exhibited significant correlations with sucrose content during fruit aging. RT-qPCR analysis demonstrated the drought stress responsiveness of *LaSUS* genes, with *LaSUS1* showing marked up-regulation under drought conditions. Furthermore, overexpression experiments in tobacco confirmed that *LaSUS1* contributed to sugar accumulation, increased antioxidant enzyme activities, and positively regulated drought tolerance in luffa. This comprehensive study not only characterizes the *LaSUS* gene family and bridges the research gap of *SUS* genes in luffa but also provides theoretical support for investigating the roles of *SUS* genes in fruit ripening and abiotic stress responses in luffa.

## 1. Introduction

Sugar, as a basic substance in plants, not only provides energy for growth and development but is also a key ingredient in many biosynthetic pathways that can be used to build carbon skeletons and generate energy [1]. The most common photosynthetic products are starch and sucrose. Sucrose is a disaccharide formed by the polymerization of glucose and fructose in a glycosidic bond and can be used as an initial energy and carbon donor for transport to other organs, especially anabolically active reservoir organs, such as plant fruits or seeds and tubers [2,3]. At the same time, sucrose also acts as a signaling molecule, regulating cell growth and development [4]. After being transported to the reservoir organs, sucrose cannot be utilized directly and needs to be hydrolyzed to glucose and fructose by invertase (INV) or reversibly catabolized to UDPG and fructose by sucrose synthase (*SUS*) [5]. It provides precursors and substrates for polysaccharide, cell wall, and starch synthesis, and it is a key enzyme in the regulation of sucrose metabolism, which is closely related to biomass formation and polysaccharide accumulation in planta [6,7]. In addition, sucrose is also involved in the senescence and ripening process of plant fruits [8]. It has been found that during the early stage of tomato fruit senescence, the content of sucrose decreases under the regulation of acid converting enzyme (AI) and *SUS*. Glucose and fructose generated by hydrolysis briefly accumulates in the early stage of senescence and then shows a decreasing trend [9]. Sucrose in strawberry accelerated the ripening process by promoting the accumulation of ABA, accompanied by changes in the content of reducing sugars [10].

Sucrose synthase (*SUS*, or called *SUS*y/SS, EC2.4.1.13), a key converting enzyme in plant sucrose metabolism, catalyzes the production of uridine diphosphate glucose (UDPG) and fructose from sucrose and uridine diphosphate (UDP), a reaction that is reversible [11]. Previous studies have found that fiberless mutants in cotton are unable to differentiate into fibers due to the lack of *SUS*y expression and the absence of bud-like protrusions in the ovule epidermis, whereas normal plants with high levels of *SUS*y expression show a promotion of fiber synthesis [12]. Overexpression of SUS in both cotton and tobacco promoted fiber elongation and significantly increased xylem cell wall thickness [13,14]. Furthermore, *SUS* also plays a role in the regulation of plant senescence. In strawberry, suppression of *FaSUS1* expression by gene silencing pushed back fruit ripening, maintained fruit firmness, and exhibited delayed anthocyanin accumulation [15]. In papaya, SUS interacts with the calcium signaling protein CML15 to regulate fruit ripening through the ABA and ethylene synergistic signaling pathway. Reduced activity of SUS delays fruit softening and reduces ethylene synthesis to delay fruit senescence [16]. In addition, previous studies have revealed that *SUS* and starch synthase (SBE) are highly expressed in pumpkin at the fruit ripening stage and regulate sugar conversion through RNA-seq [17]. Apart from its involvement in a wide range of metabolic processes, researchers have also demonstrated the role of SUS in stress response. Under low-oxygen conditions, plants adjust sucrose catabolism and tend to choose the *SUS* pathway that consumes less oxygen. *SUS* in soybean regulates sucrose metabolism and nitrogen fixation, which in turn increases the resistance of soybean to drought stress [18]. In addition, a potentially heat-resistant *SUS* was identified in wheat, which remains structurally and functionally stable at 50 °C [19].

Sugar metabolism and transport play critical roles in plant responses to abiotic stresses, by mediating osmotic adjustment and energy allocation, such as drought stress [20]. Under water deficit, plants accumulate soluble sugars (e.g., sucrose, glucose) to maintain cellular turgor and protect macromolecules, while sugar transporters facilitate their translocation to stressed tissues. Key players in this process include sucrose transporters (SUTs), hexose transporters (HTs), and trehalose-6-phosphate synthases (TPSs). For example, in rice, drought up-regulates *OsSUT1* and *OsSUT2* to enhance sucrose allocation to roots, while maize TPS genes show differential expression patterns during drought stress, contributing to trehalose biosynthesis [21]. Sucrose synthase (*SUS*), a central enzyme in sucrose metabolism, has been implicated in drought tolerance across species. In cucumber, *CsSUS3* localizes to phloem companion cells and enhances hypoxia tolerance by maintaining UDP-glucose levels for energy metabolism [22]. Similarly, overexpression of *IbSUS* in sweet potato improves nitrogen stress adaptation by regulating starch synthesis [23]. The direct studies on luffa *SUS* are limited. Functional conservation among plant species suggests analogous roles in sugar partitioning and stress resilience.

Luffa (*Luffa acutangula*), an herbaceous plant of the Cucurbitaceae family, is known for its young, tender fruits which are rich in a variety of nutrients and have a sweet flavor and health benefits. Old luffa or vines can be used for processing, as a kitchen utensil, or used in the industrial and pharmaceutical fields [24,25]. However, the systematic identification and analysis of the *LaSUS* gene family in luffa remains uncharacterized. In this study, we employed RNA-seq and bioinformatic tools, identified nine *LaSUS* family members, and systematically analyzed their physicochemical properties and evolutionary relationships. Notably, we found that *LaSUS1* plays a crucial part in drought stress response. Overall, our work fills the research gap of *SUS* genes in luffa and offers theoretical support for the *LaSUS* gene family’s functions and action mechanisms.

## 2. Results

### 2.1. Genome-Wide Identification and Characterization of LaSUS

The *SUS* gene in *Arabidopsis thaliana* was used to identify nine near-origin genes in luffa by blast (Supplemental Figure S3). Their coding DNA sequences ranged from 1131 bp in *LaSUS*4 to 3192 bp in *LaSUS*6.4. Physicochemical property analyses showed that their amino acid lengths ranged from 376 to 1063, the predicted molecular weights (MW) ranged from 44.11 to 119.28, the theoretical isoelectric points varied between 5.93 and 8.00, and the total average of hydration ranged from −0.453 to −0.043 (Table 1). To further understand the biological roles of nine putative LaSUSs, we used the online toolkit Cell-PLoc 2.0 to predict the location of the corresponding proteins in subcellular compartments, which showed that LaSUS proteins were localized in either chloroplasts or the cytoplasm (Supplemental Table S1).

### 2.2. Phylogenetic Analysis of SUS Genes in Four Cucurbitaceae Species

**Figure 1:**
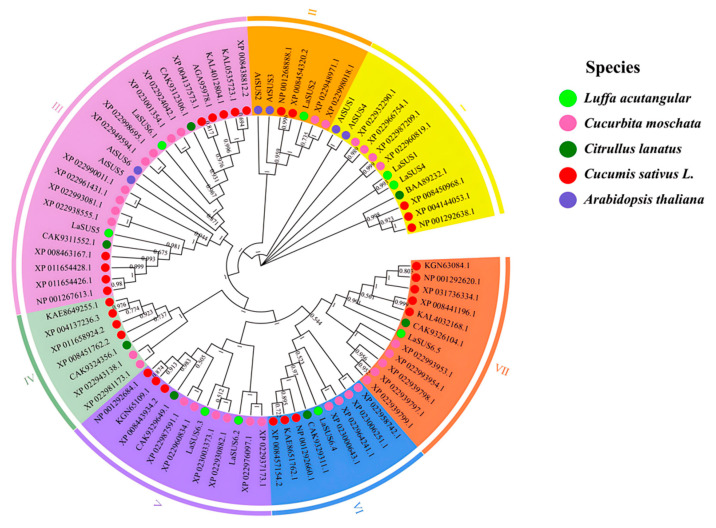
Phylogenetic analysis of *SUS* genes in four Cucurbitaceae species. Different background colors represent different groups (I to VII), the light green dots, pink dots, dark green dots, red dots, and purple dots highlight SUSs from luffa, pumpkin, watermelon, cucumber, and Arabidopsis, respectively.

Four varieties of *Luffa acutangula*, *Cucumis*, *Cucurbita*, and *Citrullus* were selected to determine the evolutionary relationship of *SUS* genes in cucurbits. The screening yielded 9, 28, 31, and 8 *SUS* members, respectively. Then, we obtained their protein sequences, including *AtSUS*, for comparison and constructed a phylogenetic tree (Figure 1). According to the branching of the phylogenetic tree, the 82 genes were clustered into seven groups (I to VII), with group III being the largest group, including 21 members of *Cucurbita*; followed by group V and group VII, both containing 12 multi-species members with complex branching, reflecting the evolutionary characteristics of the species within the *Cucurbitaceae* family. The gene sequences of *Luffa* are mainly distributed in group IV, which forms an independent branch. *LaSUS*6.2, *XP_0043137236.3*, and *XP_02281173.1* are closely aggregated, with less divergence in the evolutionary process. The group IV is farther away from the branches of other groups, which shows earlier divergence.

### 2.3. Gene Structure and Conserved Motif of LaSUS

**Figure 2:**
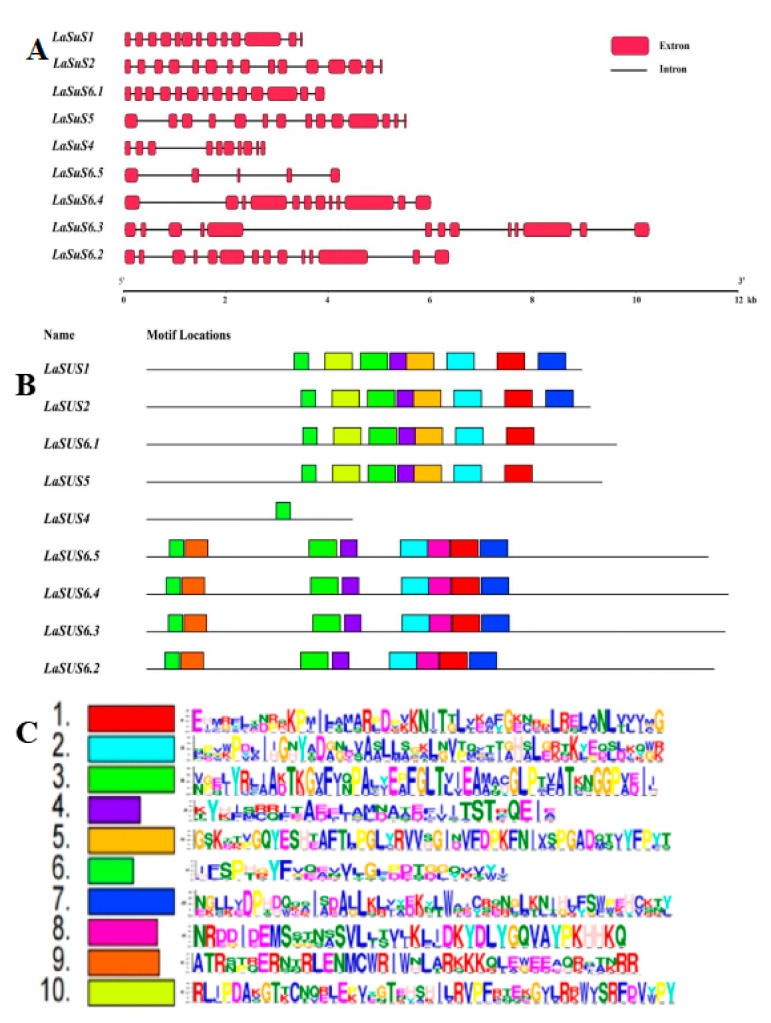
The gene structure and conserved motifs of *LaSUS*s. (**A**) Exon–intron structure of *LaSUS* genes; introns and exons are highlighted with black lines and red boxes, respectively. (**B**) The distributions of *LaSUSs* protein motifs. Each motif and their positions within each LaSUS member are represented by boxes of distinct colors. The order of the motifs corresponds to their sequential arrangement in the individual protein sequences. (**C**) Sequences of the 10 conserved LaSUS protein motifs. The different colored blocks represent sequence logos of conserved motifs.

The gene structure and the conserved motif were analyzed to further analyze the functional conservation of *LaSUS* (Figure 2A). The results showed that the candidate gene sequences covered a wide range from 0–12 kb. The number of exons varied significantly, with only 2–3 in some genes, and extra-long exons existed in some genes (*LaSUS6.4*). Meanwhile, the extrons of *LaSUS1* and *LaSUS6.1* were numerous and tightly arranged, suggesting that exon duplication events may have occurred during the evolutionary process, and the distribution of exons in *LaSUS4* and *LsSUS6.5* was more dispersed, which was speculated to have an exon deletion phenomenon, and only a small number of key functional exons were retained. The luffa SUS protein conserved motifs were analyzed using the MEME tool, and 10 conserved motifs were identified. This suggests that the *SUS* gene family has undergone structural variation during evolution, thereby contributing to functional differentiation.

The nine genes exhibit roughly two patterns of structural domains, with *LaSUS1*, *LaSUS2*, *LaSUS6.1*, and *LaSUS5* having roughly the same structural domains. A conserved structural domain was identified in *LaSUS4*, at the same location as in the four genes mentioned above. The patterns among the remaining four genes showed a high degree of similarity, which suggests their close phylogenetic relationship (Figure 2B,C).

### 2.4. Chromosomal Location and Homology Analysis of LaSUS Genes

**Figure 3:**
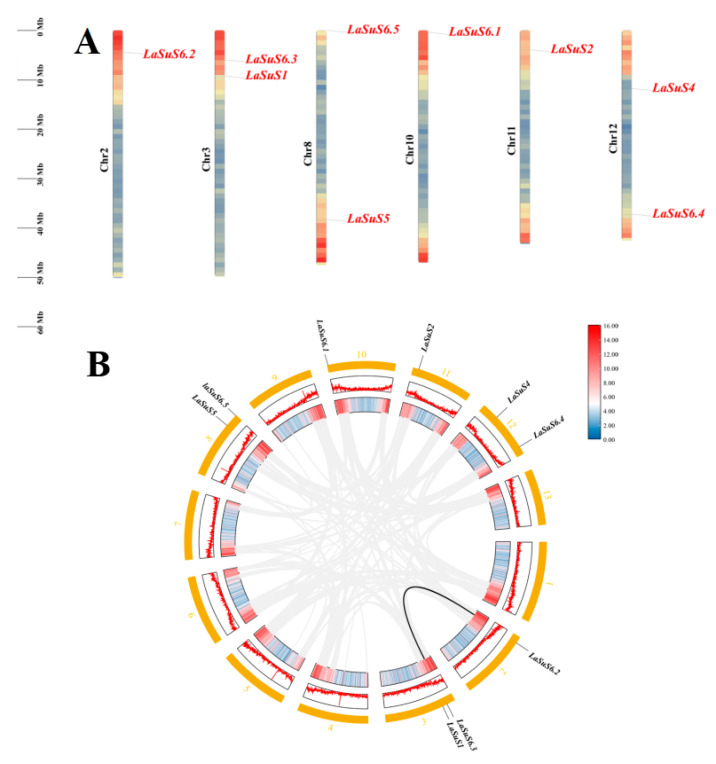
Chromosomal location and homology analysis of *LaSUS* genes. (**A**) Chromosomal localization of *LaSUS* genes. The chromosome scale in millions of bases (Mb) is shown on the left. (**B**) Co-linearity and distribution of homologous gene pairs of *LaSUS*. The numbers of each chromosome are shown outside of the circle. The gray lines represent all collinear blocks in the luffa genome, while the black lines denote gene pairs between *LaSUS* genes.

A study of the positional distribution of the nine *SUS* genes on the luffa chromosomes showed that they were unevenly distributed on six chromosomes (Figure 3A). Each of the 3.8.12 chromosomes (Chr) harbors two genes. Additionally, *LaSUS2*, *LaSUS6.2*, and *LaSUS6.3* were localized on Chr11, Chr6, and Chr2, respectively. To further characterize the evolution of the SUS family in luffa, we concentrated on gene duplication events and performed multiple covariance scans (Figure 3B). The results showed that putative segmental or tandem duplications occurred between *LaSUS6.2* and *LuSUS6.3* and *LaSUS*1, which laterally highlighted that gene duplication played a role in the evolution of the *SUS* family in luffa and contributed to the quantitative expansion of the *SUS* gene family (Supplemental Figure S4).

### 2.5. Detection of Positive Selection Among SUS Genes in Four Cucurbitaceae Species

**Figure 4:**
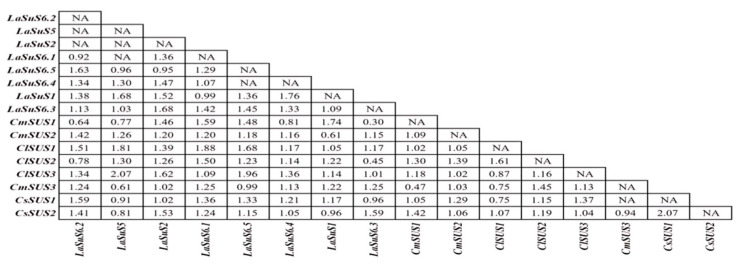
Positive selection pressure of *SUSs* between four species of Cucurbitaceae. The values in the table represent the ω values, which were estimated among *LaSUS* genes using the maximum likelihood (ML) method.

To further elucidate the evolutionary mechanism of *LaSUS* and clarify its functional dynamics, we selected several sequences with high amino acid matches, which were obtained from *Cucumis*, *Cucurbita*, and *Citrullus*, to perform collinearity analysis (Supplemental Figure S5) and pairwise ω-value analysis (Figure 4), given they are closely related with luffa. The analysis revealed that the ω values of most of the luffa SUS genes were close to or slightly higher than 1 (*LaSUS6.1* ω = 0.92, *LaSUS1* ω = 1.38) but still significantly lower than those of watermelon (ω = 2.07 for *CISUS3*) and cucumber (ω = 1.59 for *CsSUS7*), which reflected that the whole *LaSUS* family was subjected to strong purifying selection during the evolutionary process, and this may be related to the maintenance of core functions such as sugar metabolism, with low mutation probability. The high ω values of some genes (*LaSUS6.3* ω = 1.68, *LaSUS6.5* ω = 1.38) reflected the local adaptability of *LaSUS* genes.

### 2.6. Molecular Docking Reveals the Function of LaSUS in Sucrose Metabolism

**Figure 5:**
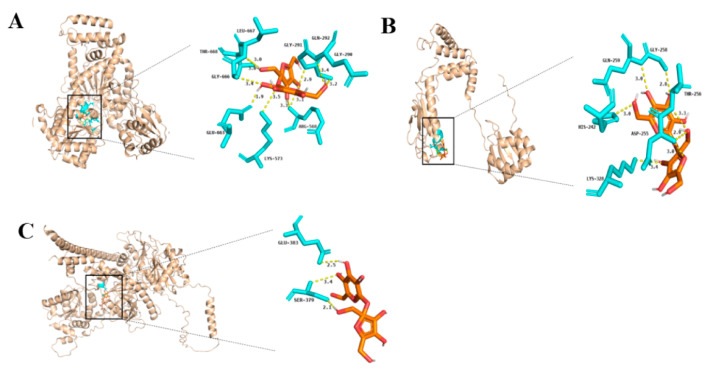
Simulated docking of LaSUSs protein with sucrose. (**A**) *LaSUS1* (type I). (**B**) *LaSUS4* (type II). (**C**) *LaSUS6.5* (type III). The orange structure represents carbon atoms, red represents oxygen atoms, white represents hydrogen atoms, and the yellow dotted line represents hydrogen bonds. The blue structure represents amino acids, and the letters on the side indicate the specific types of amino acids.

To study the protein-binding activity of LaSUS, we simulated the docking of the *LaSUS* gene and sucrose (Figure 5). Conserved motifs are important clues for the study of genes. Analysis of their sequences and distributions allows rapid speculation on the potential functions and evolutionary status of genes. Combined with the conserved motif characteristics of nine *LaSUS* genes, they can be categorized as type I (*LaSUS*1, *LaSUS*2, *LaSUS*6.1, *LaSUS*5), type II (*LaSUS*4), and type III (*LaSUS*6.5, *LaSUS*6.4, *LaSUS*6.3, *LaSUS*6.2). The similarity between genes in each type is high, so we selected one gene from each of the above three types for molecular docking, respectively. The results showed that the sucrose-binding pocket of *LaSUS*1 (type I) consisted of nine conserved amino acid residues, including THR-668, ARG-568, and GLU-663, and the free energy of binding (^Δ^G) was as high as −7.526 kcal/mol. *LaSUS*4 (type II) was roughly similar in composition to the pocket of *LaSUS*1, which had only six conserved residues with the lowest ^Δ^G. SER-379 and GLU-883 constituted the binding pocket of *LaSUS*6.5 (type III), whose ^Δ^G is second to *LaSUS*1 at −6.487 kcal/mol. High-energy binding SUS proteins (type I) may play a dominant role in the unloading and catabolism of sucrose during early filamentous fruit development, whereas low-affinity members (type II) may have a role in the long-distance transport of sucrose or the adversity response process.

### 2.7. Distribution of Cis Elements in the LaSUS Promoters

**Figure 6:**
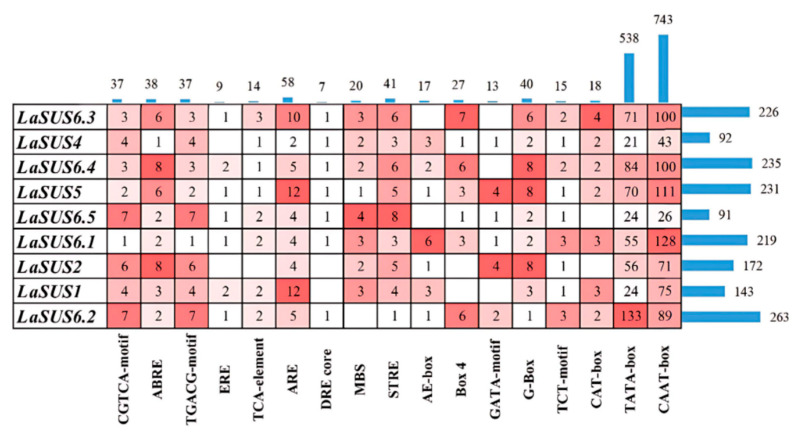
Heatmap of the number of core *cis* elements in the *LaSUSs* promoter. The colors range from deep red to light pink, representing quantities from high to low, blue columns represent the total number of individual elements or functional elements in a given gene.

Various *cis*-acting elements in promoters perform different functions. To clarify the gene function of *LaSUS*, we extracted the 2000 bp sequence upstream of the coding region as a promoter in TBtools, and then used the online website PlantCARE (https://bioinformatics.psb.ugent.be/webtools/plantcare/html/, accessed on 18 Apirl 2025) to predict the cis elements (Figure 6). The results showed that besides the core elements of the promoter (TATA-box, CAAT-box, and CAT-box), stress-responsive elements such as ARE (AAACCA) and MBS (CAACTG) were identified, suggesting that *SUS* may play a role in adverse conditions and participate in plant defense mechanisms. Hormone-responsive components such as ABRE (ACGTG) and ERE (ATTTTAAA) also appeared frequently, indicating that phytohormone content affects SUS expression levels and further regulates related physiological and metabolic processes. The number of light-responsive elements was also high, implying that SUS genes may play a role in plant photomorphogenesis and photosynthesis. Additionally, ABRE, MBS, and other components can directly regulate the expression level of sucrose transport and metabolic genes through ABA and MYB signaling pathways; ARE, CGTCA-box, and ABRE, which are related to senescence, can delay or accelerate senescence through antioxidant, JA signaling, and stress response (Supplemental Table S3).

### 2.8. Expression Levels of LaSUS in Four Postharvest Periods in Luffa Fruit

**Figure 7:**
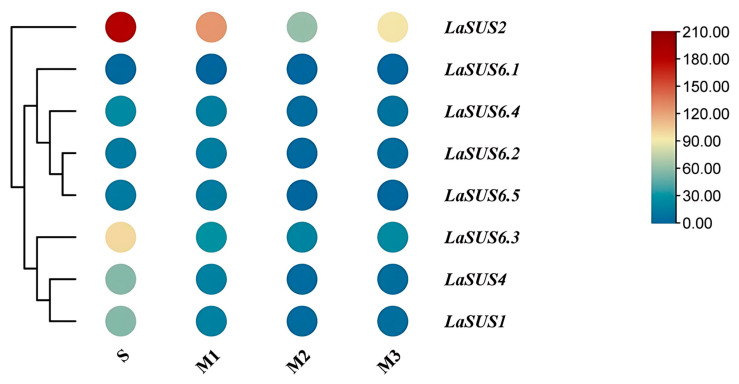
Heatmap of *LaSUS* genes FPKM expression patterns in different periods of fruit. The notations S, M1, M2, and M3 correspond to distinct stages during luffa fruit senescence; S phase represents commodity period; M1 represents 2 weeks old after S phase; M2 represents 4 weeks old after S phase; and M3 represents 6 weeks old after S phase. The color scale represents the FPKM values normalized to log_2_ (FPKM). From red to blue represents FPKM values from high to low.

We categorized the senescence progress of luffa fruits into four periods, named the S, M1, M2, and M3 phases. Then, we investigated the expression profile of these nine *SUS* genes in these four periods based on RNA-seq. Fragment per kilobase of transcript per million mapped reads (FPKM) showed that the expression of *LaSUS2* fluctuated down-regulated with the senescence of luffa fruits, but a small floating up-regulation appeared in M3 (Figure 7). *LaSUS6.3*, *LaSUS4*, and *LaSUS4* are highly expressed in the S phase and may be involved in fruit sugar accumulation metabolism during development or ripening.

### 2.9. Expression Patterns of LaSUS in Luffa Different Tissues

**Figure 8:**
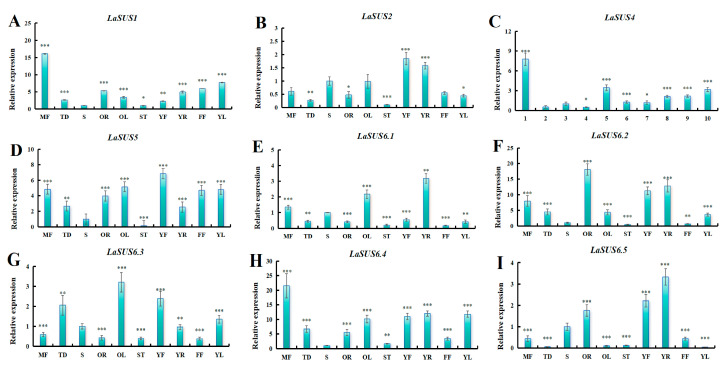
Relative transcript levels 9 *LaSUSs* in different tissues based on RT-qPCR analysis. (**A**) *LaSUS1*; (**B**) *LaSUS2*; (**C**) *LaSUS4*; (**D**) *LaSUS5*; (**E**) *LaSUS6.1*; (**F**) *LaSUS6.2*; (**G**) *LaSUS6.3*; (**H**) *LaSUS6.4*; (**I**) *LaSUS6.5*. Gene expression in the luffa samples was normalized against the expression of *LaActin*. The relative expression level for each gene was calculated with the 2^−ΔΔCT^ method. For each gene expression level visualization, the transcript level in the S (stem) tissues was set to 1.0. Different monogram represents different tissues, MF: male flower; TD; S: stem; OR: old root; OL; old leaf; ST: stem tip; YF: young flower; YR: young root; FF: female flower; YL: young leaf. Bars represent the mean value ± SD (*n* = 3) of three biological repeats. Asterisks denote statistically significant differences (* for *p* < 0.05, ** for *p* < 0.01,*** for *p* < 0.001, one-way ANOVA).

Tissue specificity may influence gene expression profiles. We investigated the expression pattern of *SUS* in different luffa tissues (Figure 8). *LaSUS*6.5 was significantly highly expressed in tissues such as young roots (YRs) and old roots (ORs). *LaSUS*6.4 was highly expressed in the reproductive tissues such as male flowers (MFs) and young fruits (YFs), which implied that it may support the reproductive organs in the process of sugar. Additionally, high expression of *LaSUS*6.2 was observed in the tendrils, demonstrating that this gene might offer energy for the rapid growth. These results suggest that there is a functional differentiation of the *SUS* family in different tissues.

### 2.10. Correlation of LaSUS Expression with Sugar and Cellulose Content

To further validate the gene function of *SUS*, we performed a correlation analysis between the *SUS* family and the sugar, and cellulose contents in the fruit during the S and M1-M3 periods (Table 2). As shown in the table, *LaSUS*1/4 (r = 0.984 *) and *LaSUS6.4* (r = 0.958 *) showed significant correlation (*p* < 0.05) with sucrose, suggesting a possible association with sucrose resynthesis, with reduced expression leading to content inhibition. However, they were not significantly correlated with cellulose content.

### 2.11. LaSUS Gene Expression Profiles Under Drought Stress

**Figure 9:**
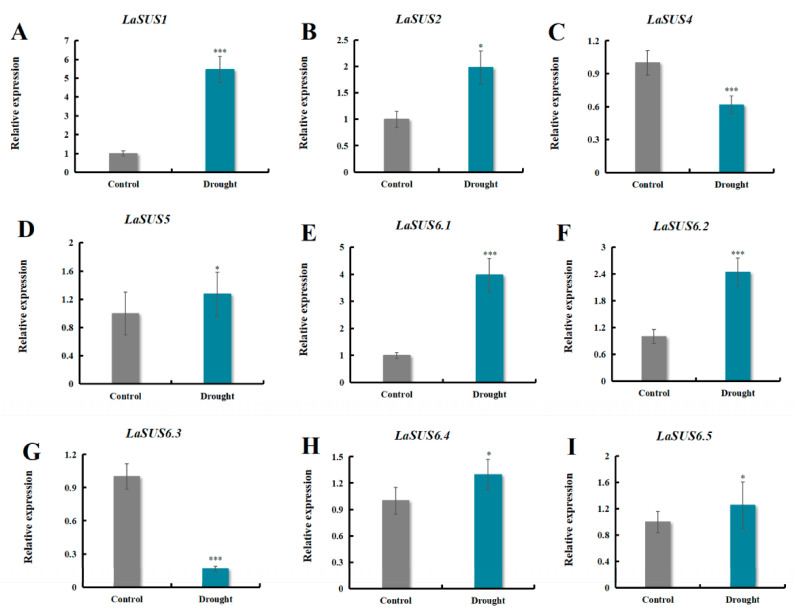
Relative transcript levels 9 *LaSUSs* in leaves under drought stress based on RT-qPCR. (**A**) *LaSUS1*; (**B**) *LaSUS2*; (**C**) *LaSUS4*; (**D**) *LaSUS5*; (**E**) *LaSUS6.1*; (**F**) *LaSUS6.2*; (**G**) *LaSUS6.3*; (**H**) *LaSUS6.4*; (**I**) *LaSUS6.5*. The transcript levels were normalized to those of *LaActin*. The control group was set to 1. Bars represent the mean value ± SD (*n* = 3) of three biological repeats. Asterisks denote statistically significant differences (* for *p* < 0.05, *** for *p* < 0.001, one-way ANOVA).

**Figure 10:**
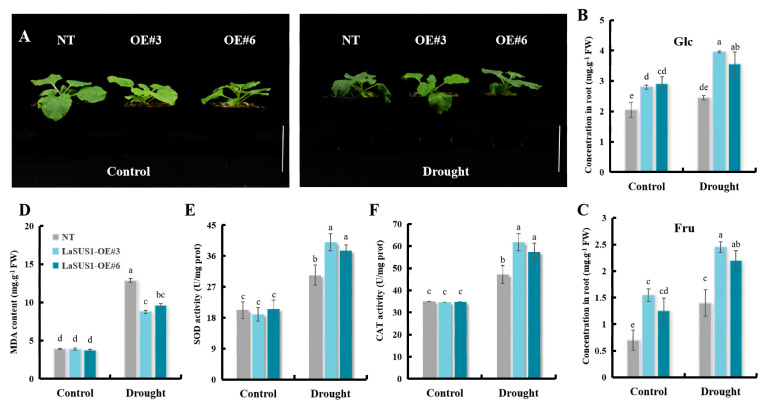
Overexpression of *LaSUS1* enhances drought tolerance in transgenic tobacco plants. (**A**) phenotypes of NT and transgenic tobacco plants overexpressing *LaSUS1* under drought stress. Scale bar, 5 cm. (**B**) Glucose (Glc); (**C**) fructose (Fru), (**D**) malondialdehyde (MDA); (**E**) superoxide dismutase (SOD); (**F**) catalase (CAT) concentrations were measured in leaf of NT and OE plants, under drought or normal growth conditions (control group). The transgenic *LaSUS* and control plants grew for 5 weeks in an incubation chamber. The drought-treated group was stopped water for 10 days, the control group watered normally. Data represent mean ± SD values of three independent biological replicates. Different alphabetical letters indicate significant differences *p* < 0.05 (one-way ANOVA).

To investigate the function of the *LaSUS* family in drought response, we determined the relative expression levels in the leaves of nine family members (Figure 9) and found that the expression of *LaSUS1* was significantly up-regulated after drought treatment. To test the effect of *LaSUS1* on drought tolerance, we generated transgenic tobacco lines overexpressing *LaSUS1* (*LaSPS1*-OE). Validation confirmed successful overexpression in T2 lines OE#3 and OE#6 (Supplemental Figure S2). Subsequently, these transgenic plants were subjected to drought stress treatment through controlled water withdrawal (10 days). The overexpression lines were able to maintain better plant morphology despite water deficit, while leaf wilting was evident in the control group (Figure 10A).

Glucose and fructose contents were significantly elevated in *LaSUS1*-overexpressed tobacco under drought conditions (Figure 10B,C). Meanwhile, the MDA content was significantly lower in the overexpression lines compared with the control (Figure 10D), suggesting that *LaSUS1* overexpression inhibited drought-induced membrane lipid peroxidation. In addition, CAT and SOD activities were higher in *LaSUS1* overexpressed tobacco compared with NT under drought stress, suggesting that *LaSUS1* could regulate sucrose metabolism and improves antioxidant capacity under drought stress (Figure 10E,F). This is consistent with the mechanism of *TaSUS2* in wheat in response to drought stress [26].

## 3. Discussion

Photosynthesis is the main pathway for carbon sequestration in plants. Sucrose is a key product of this process and the main sugar that is transported from the source tissues to the reservoir tissues through the phloem [27]. Sucrose synthase (*SUS*), a glycosyltransferase, is involved in the reversible catabolic synthesis of sucrose and has an effect on plant sugar metabolism [28]. Previously, *SUS* genes have been identified in several species and mostly in the form of small gene families. For example, five members were identified in grape (*Vitis vinifera*) and sugarcane (*Saccharum* spp.) [29]. There are seven *SUS* homologous genes were identified in apple (*Malus domestica*) [30]. Additionally, the number was higher in tobacco, poplar, and Chinese pear, which were 14, 15, and 30, respectively [31,32,33]. However, at present, there is a lack of systematic analysis of *SUS* family evolution, expression patterns, and co-regulatory mechanisms in luffa. The situation that encourages us to target *LaSUS* and to investigate its physicochemical properties, gene functions, and evolutionary features.

The visualization of nine *LaSUS*s structures revealed that the family members all contained typical exon–intron structures, but there were significant differences among them. The exon numbers of *LaSUS*1 subfamily members (*LaSUS*1, *LaSUS*2, *LaSUS*5, *LaSUS*6.1, *LaSUS*6.2) were highly conserved, with the number of exons being >13, which provided similarity in the key functions of the genes providing evidence [34]. In addition., longer introns appeared in *LaSUS*6.3, *LaSUS*6.4, and *LaSUS*6.5. This characteristic demonstrates that they may generate transcripts by mutation or shearing to adapt to environmental changes [35]. Motif analysis results indicate that the *LaSUS* gene family has a common domain (shown in the green box), which is associated with the sucrose synthase function. It is hypothesized that this domain may represent the core functional domain of *SUS*.

The nine members of the luffa SUS gene family are unevenly distributed on six chromosomes, with two *LaSUS* members each on chromosomes 3, 8, and 12. SUS family evolutionary analysis was performed in combination with three other cucurbit species: cucumber (*Cucumis sativus* L.), watermelon (*Citrullus lanatus*), and pumpkin (*Cucurbita moschata*). Interspecific covariance analysis of the above species showed that the pairwise probability of matching between SUS genes was high in luffa and pumpkin and cucumber, implying the occurrence of a whole genome duplication event (WGD) between them, whereas the *SUS* genes of watermelon diverged earlier. Meanwhile, the results of selection evolutionary pressure analysis demonstrated that the overall SUS genes of luffa were subjected to purifying selection (dN/dS < 1), presenting a functionally conserved phenotype, retaining the key functions in sucrose catabolism metabolism. However, *LaSUS*6 subfamilies (e.g., *LaSUS*6.2/6.3) have dN/dS values close to 1, suggesting that they may have undergone functional divergence during evolution to meet physiological demands [36]. Compared with Arabidopsis (*Arabidopsis thaliana*), cucurbit *SUS* genes form independent branches in the evolutionary tree, reflecting the functional differentiation between monocotyledons and dicotyledons in the regulation of sugar metabolism [37].

There are various types of *cis* elements in the *SUS* family, such as ABRE, ERE, CAAT-box, etc. *LaSUS6.3* has the highest number of six ABRE elements. ABRE plays a role in plant response to stress, implying that the *LaSUS* family changes the expression level to adapt resistance (e.g., drought, high temperature, etc.). The diverse range of *cis* elements illustrates the involvement of the *SUS* family in various physiological activities during the growth and development of luffa. The expression levels of *SUS* genes in different tissues were measured by RT-qPCR. The expression level of *LaSUS6.5* in young roots (YRs) was 164-fold higher than in young leaves (YLs). Studies in maize and other crops demonstrate that elevated expression of *SUS* genes in roots correlates strongly with enhanced root vigor and carbon assimilate partitioning [38]. As YRs undergo rapid growth and differentiation, requiring substantial nutrient and energy resources, *SUS* likely facilitates these metabolic processes. Concurrently, *LaSUS2* exhibited high expression during the young fruit (YF) stage, potentially supporting carbohydrate biosynthesis and accumulation critical for fruit development. These genes represent promising functional research targets warranting further investigation.

Moreover, we observed a decreasing trend of *SUS* expression level in four postharvest periods (S, M1, M2, and M3) of luffa fruits, which might be caused by the massive expression of ABA and ethylene during senescence. In Arabidopsis, ABA treatment down-regulated the expression of the *AtSUS3* gene by 60% [39], and ethylene was synthesized in large quantities during banana post-ripening, which specifically binds to the ERE element in the promoter region of the SUS gene and represses the transcription [40]. In addition, at the postmaturity stage, cell entry into programmed death down-regulates transcript abundance [39], and the accumulation of reactive oxygen species accelerates the degradation of SUS proteins [40], which may be the triggers for the reduction of SUS expression level. Strangely, along with the down-regulation of SUS expression, the sucrose also continued to decrease, and their expression levels presented a significant positive correlation in some genes (*LaSUS*1, r = 0.984 *; *LaSUS*4, r = 0.984 *). The decrease in sucrose may be the result of a shift to sucrose catabolism as the cells enter senescence to provide glucose for the synthesis of the cellulose precursor UDPG. This process may be associated with sucrose convertase INV and sucrose phosphate synthase (SPS).

Drought stress impairs seed germination, plant growth, and yield, causing direct or indirect damage to plants [41]. Drought treatment significantly induced the expression of several *LaSUS* members. Among them, *LaSUS1* showed the most significant expression. We verified the function of *LaSUS1* in drought response. Overexpression of *LaSUS1* in tobacco exhibited enhanced drought tolerance accompanied by increased Glc and Fru content, suggesting that *LaSUS1* may affect osmosis by regulating sucrose metabolism to improve drought tolerance.

Additionally, *SUS* plays an important role in the non-biological response processes of other species by dynamically regulating sucrose metabolism to help plants adapt to stresses such as drought, high temperatures, and waterlogging. For instance, the expression of the rice gene *OsSUS2* increases under low-oxygen stress, promoting sucrose breakdown to maintain the supply of energy [42]. Meanwhile, the expression of the Populus *PtSUS2* increases significantly under drought stress, enhancing cellulose synthesis in cell walls to improve mechanical strength [43]. The expression level of the passion fruit *PeSUS5* gene increases significantly under high-temperature stress, while *PeSUS1* and *PeSUS2* are down-regulated, indicating their differential regulation in response to high temperatures [44].

## 4. Materials and Methods

### 4.1. Identification of LaSUS and Phylogenetic Analysis

The amino acid sequences of Arabidopsis *SUS* genes were obtained from The Arabidopsis Information Resource (TAIR) (https://www.arabidopsis.org/, accessed on 13 March 2025, and 9 SUS family genes were obtained by blast in the luffa self-constructed luffa database using the software TBtools (version 2.308) [45]. The physicochemical parameters of *LaSUS*, such as molecular weight (MW), theoretical isoelectric point (PI), instability index, and aliphatic index, were analyzed on https://web.expasy.org/protparam/ (accessed on 2 Apirl 2025). In addition, these 9 genes were blasted in the NCBI (National Center for Biotechnology Information) website, and 76 genes with high relevance in cucurbita, citrullus and cucumis were screened. Then, we downloaded protein sequences for sequence comparison and used the NJ method to construct a phylogenetic tree in MEGA 11.0 software.

### 4.2. Chromosomal Mapping and Syntenic Analysis of LaSUS

The chromosomal localization information of these 9 *SUS* genes was obtained from the luffa database, and the data information was imported into TBtools (version 2.308) for visualization. The information of the Luffa genome and the IDs were obtained to perform the covariance analysis in TBtools.

### 4.3. Gene Structure, Conserved Motifs, and Subcellular Localization Analysis of LaSUS

Virtualization of gene structures was performed using TBtools (version 2.308). We utilized the online MEME Suite server (http://meme-suite.org/, accessed on 27 March 2025) for motif analysis and screened 10 conserved structural domains, while subcellular localization was performed using the online service package Cell-PLoc 2.0 (Supplemental Table S1) [46,47,48,49,50].

### 4.4. Cis Element Screening in the Promoters

We extracted the 2000 bp upstream sequences of the *SUS* genes’ coding region, retrieved as promoter sequences with the help of TBtools (version 2.308). The 9 gene sequences’ information was integrated and analyzed using the online tool PlantCARE (https://bioinformatics.psb.ugent.be/, accessed on 18 Apirl 2025) to identify possible cis-acting elements. After organizing the data, we made a heat map in Microsoft Excel 365 (Version 2211, Microsoft Corporation, Redmond, DC, USA) to show the number of components.

### 4.5. Selective Pressure Analysis of LaSUS

The coding sequences of the *SUS* genes of four cucurbits were compared to the sequences using MEGA (version 12.0), and the evolutionary tree was constructed by the NJ method to obtain nwk files. The above files were imported into PamlX (version 2.1). Then, we performed two-by-two comparisons using the CodeML component according to the maximum approximation method and output the ω value (dN/dS ratio).

### 4.6. Molecular Docking

Firstly, the CAS number of small-molecule sucrose was obtained through a web search; then, we downloaded the PDB file of the sucrose reference model from NCBI-PubChem (https://pubchem.ncbi.nlm.nih.gov/, accessed on 13 Apirl 2025). The structure prediction of the large molecule SUS was realized in the Website SWISS-MODEL (https://swissmodel.expasy.org/, accessed on 14 Apirl 2025). After inputting the amino acid sequences of the genes, the model with the highest GMQE value was selected, and the PDB file was downloaded. Next, the two models were pre-processed with the help of AutoDock (version 1.5.7), including hydrogenation, water removal, and charge calculation. After completing the above operations, the model files were saved in PDBQT format, and AutoDock-vina was run to perform molecular docking, exported to PDB format, and visualized in Pymol software (version 3.1).

### 4.7. The Expression Level Measurement of LaSUS

Fruits were sampled at four periods, the S phase (Storage period), M1 (2 weeks old after storage period), M2 (4 weeks old after storage period), and M3 (6 weeks old after storage period). The tissues of luffa included 10 parts, male flowers (MFs), female flowers (FFs), young leaves (YLs), middle leaves (OLs), young stems (STs), middle stems (Ss), young roots (YRs), old roots (ORs), young fruits (YFs), and tendrils (TDs). Total RNA from each part was extracted according to previous descriptions [51]. Next, 1 μg RNA was reverse-transcribed to cDNA with Thermo Scientific’s RevertAid M-MuLV (Thermofisher Scientific accompany, Shanghai, China). Each RT-qPCR experiment included three biological replicates, with each biological replicate containing 3 technical repeats. The reference conditions for RT-qPCR were as follows: initial denaturation at 95 °C for 30 s, followed by 40 cycles of amplification at 95 °C for 5 s and 60 °C for 20 s. Following amplification, a melting curve analysis was performed by gradually increasing the temperature from 65 °C to 95 °C with continuous fluorescence measurement to confirm the specificity of the PCR products and the absence of primer dimers or non-specific amplification. Representative melting curves demonstrating product specificity are provided in Supplemental Figure S1. Gene expression in the tobacco samples was normalized against the expression of the *NtActin* (AB158612) gene as previously described [52]. For luffa, against that of *LaActin* (LacutCM022711.1G022380.1). All primers used in this study are listed in the Supplemental Table S2.

### 4.8. Construction of LaSUS Transgenic Tobacco Plants

The CDS (coding sequence) sequences of *LaSUS1* genes were cloned and embedded in a constructed overexpression vector (*p*CAMBIA2300, CAMBIA), which is driven by the enhanced CaMV 35S promoter. Tobacco was transformed as described previously [53,54]. The transgenic tobacco seeds were then surface sterilized with 0.2% NaClO, stratified at 4 °C for several days, and grown on selective agar medium. Transgenic tobacco lines harboring *pCAMBIA2300-LaSUS1* were selected on kanamycin (50 mg/L) containing medium. Following rooting and acclimatization, verified plants were transferred to a greenhouse and cultivated until flowering. Integration of *LaSUS1* transgene was confirmed by genomic PCR and RT-qPCR analyses (Supplemental Figure S2). T2 generation transgenic plants were utilized for subsequent experiments.

### 4.9. Drought Stress Treatments and Physiological Indicators Measurements

We planted seedlings of transgenic *LaSUS* and control plants grew for 5 weeks in an incubation chamber at 30 °C (16 h light/8 h dark cycle). During drought experiment, plants (10 individuals per line) were divided into a control group and a drought-treated group. For the drought-treated group, watering was terminated when the soil relative water content (RWC), measured using a soil moisture meter (TDR350, Spectrum America Inc., Boca Raton, FL, USA), consistently reached ≥70%. Subsequently, a 10-day water withholding period was imposed to induce drought stress. We collected transgenic plant tissues (OE#3 and OE#6) and determined the MDA content [55], glucose content, and fructose content [56]. The CAT and SOD activity were measured using specific detection kits according to the manufacturers’ instructions (Suzhou Comin Biotechnology Co., Ltd., Suzhou, China).

### 4.10. Statistical Analysis

All data analysis was conducted using SPSS Statistics 21 (SPSS, Inc., Chicago, IL, USA), while data visualization was performed with Microsoft Excel 365 (Version 2211, Microsoft Corporation, Redmond, United States of America). Statistical analysis employed one-way analysis of variance (ANOVA), with statistical significance defined as *p* < 0.05. Reported values represent the mean ± standard deviation (SD) from three independent experimental replicates.

## 5. Conclusions

This study systematically identified and characterized nine *LaSUS* gene family members in luffa. Evolutionary analyses, chromosomal localization, and covariance analyses were also performed on these family members. The expression profiles in different tissues indicated that *LaSUS* mediated sucrose metabolism may be involved in several physiological processes in luffa. Additionally, we noticed a special phenomenon that sucrose content was synchronously down-regulated with *LaSUS* expression level during luffa fruit senescence. Then, we made reasonable inferences from the experimental data and previous research results. Also, *LaSUS* candidate genes involved in drought stress were identified, and *LaSUS*1 could alleviate reactive oxygen species damage by maintaining osmotic balance. In summary, our study provides a theoretical reference for understanding the evolutionary mechanism and biological role of the *LaSUS* gene family in luffa.

## Figures and Tables

**Table 1:** Summary of biological and physicochemical properties of LaSUS proteins. CDS, coding sequence; MW, molecular weight; pI, theoretical isoelectric point; GRAVY, grand average of hydropathicity.

Gene Name	Gene ID	CDS	Chromosome Location	Amino Acids	MW (kDa)	PI	GRAVY	Instability Index	Aliphatc Index
*LaSUS1*	*LC_Maker00039001_R1*	2392	Chr3	796	91.63	5.95	−0.29	30.86	93.19
*LaSUS2*	*LC_Maker00039001_R0*	2436	Chr11	811	92.44	6.02	−0.221	34.62	94.35
*LaSUS6.1*	*LC_Maker00036130_R0*	2580	Chr10	859	97.28	7.57	−0.341	43.17	85.72
*LaSUS5*	*LC_Maker00008755_R0*	2499	Chr8	832	94.45	6.24	−0.292	38.16	85.32
*LaSUS4*	*CS_CsGy1G028230.2_R4*	1131	Chr12	376	44.11	6.71	−0.246	29.17	103.38
*LaSUS6.5*	*LC_Maker00030741_R0*	3081	Chr8	1026	115.76	6.44	−0.440	42.36	89.42
*LaSUS6.4*	*CS_CsGy2G009040.2_R0*	3192	Chr12	1063	119.28	6.05	−0.453	45.36	85.42
*LaSUS6.3*	*CS_CsGy1G017180.1_R0*	3174	Chr3	1057	117.29	6.04	−0.382	41.66	87.38
*LaSUS6.2*	*LC_Maker00039364_R2*	3114	Chr2	1037	115.73	5.93	−0.375	40.31	88.36

**Table 2:** The correlation coefficient of the *LaSUSs* with the expression of glucose, fructose, sucrose, and cellulose at four postharvest periods in luffa fruit. (One asterisk represent significant differences at *p* < 0.05 (one-way ANOVA).)

	Fru	Glu	Suc	Cellulose
*LaSUS1*	0.865	0.855	0.984 *	−0.749
*LaSUS2*	0.886	0.808	0.945	−0.376
*LaSUS4*	0.865	0.855	0.984 *	−0.690
*LaSUS6.1*	0.469	0.507	0.747	−0.935
*LaSUS6.2*	0.941	0.849	0.811	−0.806
*LaSUS6.3*	0.768	0.766	0.942	−0.749
*LaSUS6.4*	0.928	0.856	0.958 *	−0.857
*LaSUS6.5*	0.969 *	0.896	0.852	−0.645

## Data Availability

All relevant data are included within the paper and its Supplementary Materials.

## Supplementary Materials

The following supporting information can be downloaded at: https://www.mdpi.com/article/10.3390/ijms26125675/s1.
