# Supplementary material for: Genome-Wide Identification of Luffa Sucrose Synthase Genes Reveals LaSUS1-Mediated Sugar Metabolism Boosting Drought Tolerance
Source: Int J Mol Sci. 2025 Jun 13;26(12):5675. doi: 10.3390/ijms26125675 (PMC12192859; doi:10.3390/ijms26125675)
Supplement: Supplementary file 1 [file ijms-26-05675-s001.zip › Supplemental Figures.pdf]

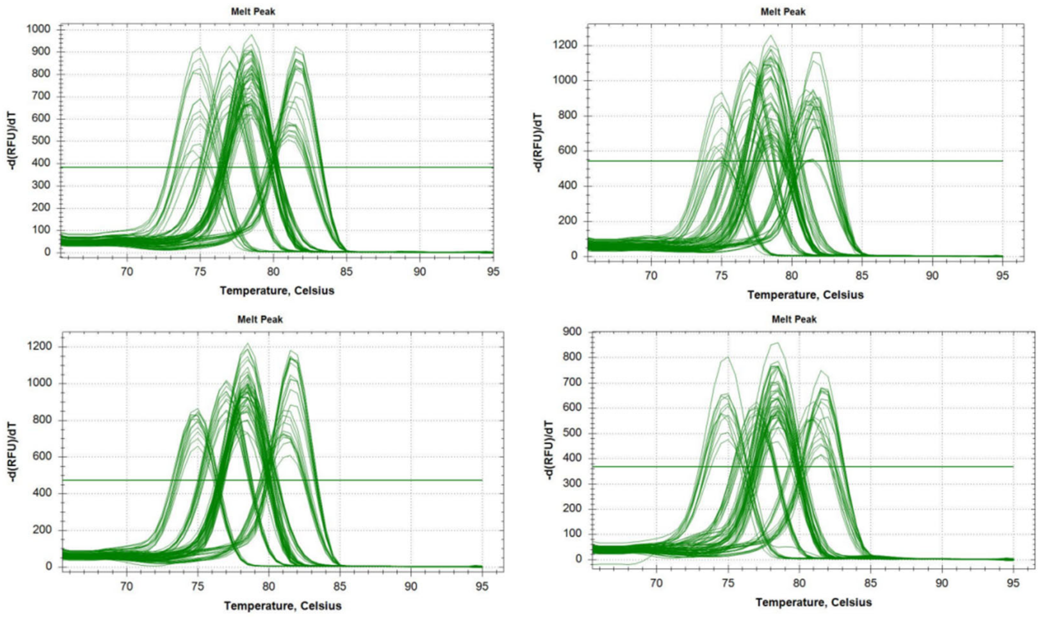

Supplemental Fig. S1 Melting curves for RT-qPCR analysis. Representative melting curve plots demonstrating a single sharp peak for each amplicon, confirming the specificity of the amplification. This lysis curve demonstrates the genes that appear in Figures 8 and 9.

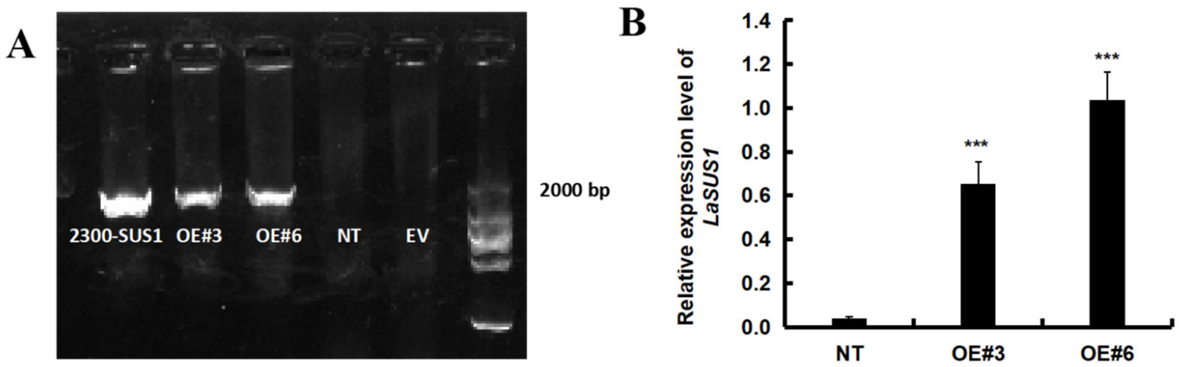

Supplemental Fig. S2 Successful integration of the LaSUS1 transgene in transgenic lines OE#3 and #6 was confirmed by genomic PCR and RT-qPCR analysis.

A, Genomic PCR analysis confirmed the successful expression of LaSUS1 in tobacco lines OE#3 and OE#6. The recombinant plasmid 2300-LaSUS1 served as the positive control, while non-transgenic (NT) and empty vector (EV) served as negative controls.

B, RT-qPCR analysis of LaSUS1 transcript levels in transgenic tobacco lines OE#3 and OE#6. Bars epresent the mean value ± SD (n=3) of three biological repeats. Asterisks denote statistically significant differences (*** for $p < 0.001$, one-way ANOVA).

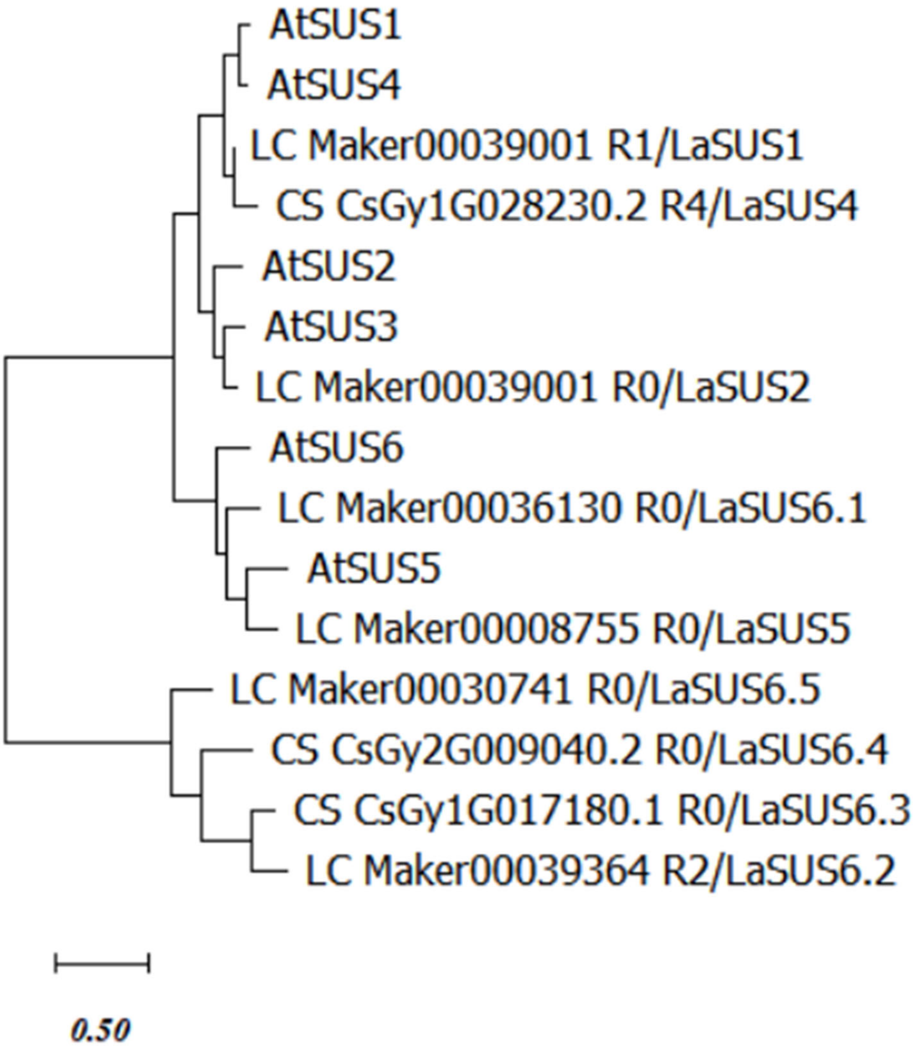

Supplemental Fig. S3 Analysis of the evolutionary relationship between luffa and *Arabidopsis thaliana*.

	LaSuS6.2	***LaSuS5***	***LaSuS2***	***LaSuS6.1***	***LaSuS6.5***	***LaSuS6.4***	***LaSuS1***	***LaSuS6.3***
LaSuS6.2	NA							
LaSuS5	NA	NA						
LaSuS2	NA	NA	NA					
LaSuS6.1	0.92	NA	1.36	NA				
LaSuS6.5	1.63	0.96	0.95	1.29	NA			
LaSuS6.4	1.34	1.30	1.47	1.07	NA	NA		
LaSuS1	1.38	1.68	1.52	0.99	1.36	1.76	NA	
LaSuS6.3	1.13	1.03	1.68	1.42	1.45	1.33	1.09	NA

Supplemental Fig. S4 Positive selection pressure analysis of luffa.

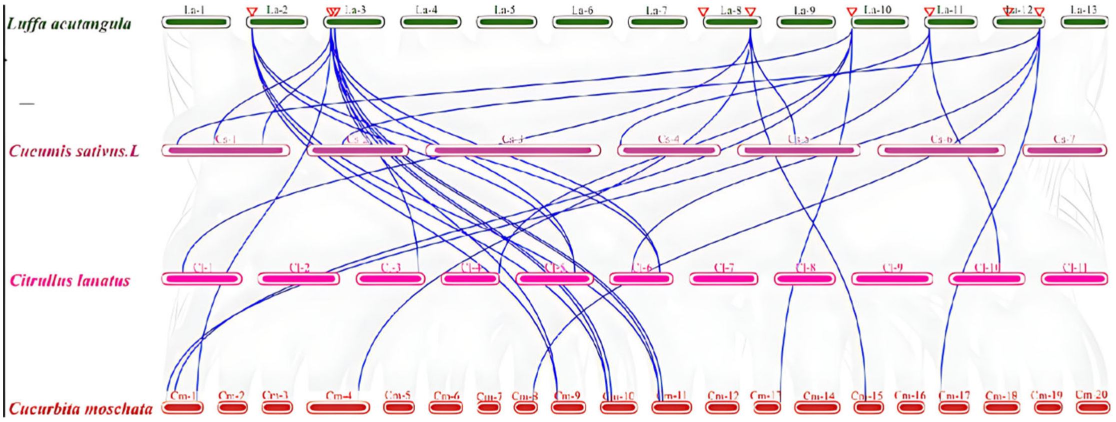

Supplemental Fig. S5 Co-linearity of *SUS* among four Cucurbitaceae species.
