# Supplementary material for: Genome-Wide Identification of Luffa Sucrose Synthase Genes Reveals LaSUS1-Mediated Sugar Metabolism Boosting Drought Tolerance
Source: Int J Mol Sci. 2025 Jun 13;26(12):5675. doi: 10.3390/ijms26125675 (PMC12192859; doi:10.3390/ijms26125675)
Supplement: Supplementary file 1 [file ijms-26-05675-s001.zip › Supplemental Table S1.pdf]

Supplemental Table S1. Subcellular localization of LaSUSs.

	Cyt	Pla	Mit	Per	Chlt	Nuc	ER	Gol	Extra	Cyt	Ly	Vac
LaSUS1	3.492*	0.283	0.271	0.239	0.237	0.192	0.102	0.074	0.071	0.023	0.011	0.007
LaSUS2	2.373	0.590	0.761	0.447	0.403	0.223	0.083	0.035	0.037	0.019	0.013	0.014
LaSUS4	3.141	0.124	0.43	0.296	0.042	0.591	0.094	0.154	0.104	0.010	0.008	0.006
LaSUS5	3.335	0.151	0.435	0.276	0.281	0.344	0.039	0.030	0.069	0.010	0.012	0.017
LaSUS6.1	2.567	0.182	0.937	0.293	0.244	0.548	0.062	0.042	0.072	0.021	0.010	0.020
LaSUS6.2	1.803	0.129	0.446	0.1852	0.411	1.667*	0.057	0.187	0.046	0.028	0.022	0.023
LaSUS6.3	1.292*	0.179	0.528	0.134	1.075*	1.502*	0.050	0.152	0.026	0.031	0.015	0.018
LaSUS6.4	1.227	0.090	0.490	0.049	0.230	2.689*	0.033	0.121	0.024	0.032	0.005	0.009
LaSUS6.5	1.335	0.294	0.418	0.05	0.387	2.307*	0.046	0.088	0.041	0.015	0.008	0.011

Cyt represents Cytoplasmic, Pla represents Plasma Membrane, Mir represents Mitochondrial, Per represents Peroxisomal, Chlt represents Chloroplast, Nuc represents Nuclear, Gol represents Golgi, Extra represents Extracellular, Cyt represents Cytoskeletal, Ly represents Lysosomal, Vac reresents Vacuole.
