# Supplementary material for: Genome-Wide Identification of Luffa Sucrose Synthase Genes Reveals LaSUS1-Mediated Sugar Metabolism Boosting Drought Tolerance
Source: Int J Mol Sci. 2025 Jun 13;26(12):5675. doi: 10.3390/ijms26125675 (PMC12192859; doi:10.3390/ijms26125675)
Supplement: Supplementary file 1 [file ijms-26-05675-s001.zip › Supplemental Table S2.pdf]

Supplemental Table S2. Primer used in this study

Primer Name	Sequence(5'-3')	Primer annotation
2300-LaSuS1-KpnI-F	CGGGGGACGAGCTCGGTACCATGGCAGAACGAGTCCTCAA	Gene clone
2300-LaSuS1-XbaI-R	CACCATGGTGTCGACTCTAGACTCGTCCTCAGCCAGTGG	
qPCR-LaSuS6.2-F	GTGATTCCGATACTGGTG	RT-qPCR
qPCR-LaSuS6.2-R	GCCAAAGAAGTTTCTCCC	
qPCR-LaSuS5-F	TTGGGAAGTTTGTGGAG	
qPCR-LaSuS5-R	CTTAGGGCAAAGGCAAC	
qPCR-LaSuS6.1-F	AAGATTCGGATGCTAAATG	
qPCR-LaSuS6.1-R	AGTCCAGGCATAGTAAAGG	
qPCR-LaSuS2-F	AAGCAACTGAGGCTTACA	
qPCR-LaSuS2-R	AACCACCAACAACAACTAG	
qPCR-LaSuS4-F	TGTTCACAGCCTCCACG	
qPCR-LaSuS4-R	CTTCGCCAAATGTCTCA	
qPCR-LaSuS1-F	CCTCAACCGTCACCTCT	
qPCR-LaSuS1-R	CCCACCCTCTAAACTCG	
qPCR-LaSuS6.3-F	AGAGCAGTGGTGCCTATA	
qPCR-LaSuS6.3-R	GCCAGACTGGATGTCCC	
qPCR-LaSuS6.5-F	GGACCTCGTGATAAATACA	
qPCR-LaSuS6.5-R	ACAAGTGAGCAGCCACC	
qPCR-LaSuS6.4-F	TGAATCGGACCTTCACC	
qPCR-LaSuS6.4-R	CCAATCTCCGGTTTGTG	
NtACTIN-F	TCCTGATGGGCAAGTGATTAC	
NtACTIN-R	TTGTATGTGGTCTCGTGGATTC	

LaActin-F	GTACAACTGGTATCGTGCTG	
LaActin-R	AGGTCCAAACGGAGAATT	
