# Supplementary material for: Genome-Wide Identification of Luffa Sucrose Synthase Genes Reveals LaSUS1-Mediated Sugar Metabolism Boosting Drought Tolerance
Source: Int J Mol Sci. 2025 Jun 13;26(12):5675. doi: 10.3390/ijms26125675 (PMC12192859; doi:10.3390/ijms26125675)
Supplement: Supplementary file 1 [file ijms-26-05675-s001.zip › Supplemental Table S3.pdf]

Supplemental Table S3. The information of core cis-elements.

Module	Core sequence	Functions
CGTCA-motif	CGTCA	cis-acting regulatory element involved in the MeJA-responsiveness
TGACG-motif	TGACG	cis-acting regulatory element involved in the MeJA-responsiveness
ABRE	ACGTG	cis-acting regulatory element involved in ABA responsiveness
TCA-element	TCAGAAGAGG/CCATCTTTTT	cis-acting element involved in salicylic acid responsiveness
ERE	ATTTTAAA	cis-acting regulatory element involved in hormone
ARE	AAACCA	cis-acting regulatory element essential for the anaerobic induction
DRE core	GCCGAC	cis-acting regulatory element involved in abotic stress or binding with DREB-like transcription factors
MBS	CAACTG	MYB binding site involved in drought-inducibility
STRE	AGGGG	cis-acting regulatory element involved in abotic stress
AE-box	AE-box	part of a module for light response
BOX 4	ATTAAT	part of a conserved DNA module involved in light responsiveness
GATA-motif	GATAGGA/AAGATAAGATT	part of a light responsive element
TCT-motif	TCTTAC	part of a light responsive element
G-box	CACGT[G/T/A]	cis-acting regulatory element involved in light responsiveness
TATA-box	TATA	core promoter element around -30 of transcription start
CAAT-box	CAAAT/CAAT	common cis-acting element in promoter and enhancer regions
CAT-box	GCCACT	cis-acting regulatory element related to meristem expression
